# The age-related posterior-anterior shift as revealed by voxelwise analysis of functional brain networks

**DOI:** 10.3389/fnagi.2014.00301

**Published:** 2014-11-07

**Authors:** Paul McCarthy, Lubica Benuskova, Elizabeth A. Franz

**Affiliations:** ^1^Department of Computer Science, University of OtagoDunedin, New Zealand; ^2^Nuffield Department of Clinical Neurosciences, FMRIB Centre, University of OxfordOxford, UK; ^3^Brain Health Research Centre, University of OtagoDunedin, New Zealand; ^4^Department of Psychology, University of OtagoDunedin, New Zealand; ^5^fMRIOtago, University of OtagoDunedin, New Zealand

**Keywords:** PASA, Alzheimer's disease, fMRI, functional connectivity, functional network analysis, graph theory

## Abstract

The posterior-anterior shift in aging (PASA) is a commonly observed phenomenon in functional neuroimaging studies of aging, characterized by age-related reductions in occipital activity alongside increases in frontal activity. In this work we have investigated the hypothesis as to whether the PASA is also manifested in functional brain network measures such as degree, clustering coefficient, path length and local efficiency. We have performed statistical analysis upon functional networks derived from a fMRI dataset containing data from healthy young, healthy aged, and aged individuals with very mild to mild Alzheimer's disease (AD). Analysis of both task based and resting state functional network properties has indicated that the PASA can also be characterized in terms of modulation of functional network properties, and that the onset of AD appears to accentuate this modulation. We also explore the effect of spatial normalization upon the results of our analysis.

## 1. Introduction

A number of imaging and behavioral studies suggest that older adults have reduced activity compared to younger adults in occipitotemporal regions but increased activity in anterior regions. Davis et al. (2008) named this phenomenon the Posterior-Anterior Shift in Aging (PASA) and showed that it reflects the effects of aging rather than differences in task difficulty, and thus represents a general phenomenon of brain aging. In the present work we aim to investigate the PASA phenomenon from the point of view of graph-theoretical analysis of voxelwise functional brain networks (Sporns, 2011). In addition we extend the analysis to assess whether the PASA pattern would be accentuated or altered with the onset of Alzheimer's Disease (AD).

Functional MRI is often used to learn about how the attributes of a particular cohort of individuals affect brain function, and a wide body of work has been undertaken exploring the potential for attributes of fMRI data to act as markers for various neurological disorders and diseases (e.g., Achard et al., 2012; Zhao et al., 2012; Jacobs et al., 2013; Seo et al., 2013). Alzheimer's Disease (AD) has received particular attention due to its increasing prevalence amongst a global increase in elderly populations. AD is an irreversible form of dementia characterized by progressive deterioration of both intellect and behavior, and has a substantial social and economic impact upon sufferers, their families, and society as a whole. AD has been described as *the coming plague of the 21st century* (Mandell and Green, 2011). The discovery of a definitive method for the early diagnosis of AD is of crucial importance as, by the time any cognitive and behavioral decline becomes clinically observable in a sufferer, pathological atrophy is already substantial (Killiany, 2011).

The analysis of functional connectivity involves quantifying the strength of temporal correlations, or functional links, between different predefined parts of brain tissue (Friston et al., 1993). The links that are found form a network of interaction which allows inferences to be made about the higher level structure, or complexity, of patterns of brain connections, something that standard methods of functional imaging analysis typically do not allow (Bullmore and Sporns, 2009; Rubinov and Sporns, 2010; Sporns, 2011). Whilst still very much a technique under development, functional network analysis can provide new insights into neurological function and behavior, and should be seen as a complement to more traditional approaches for the study of functional activation and connectivity, such as the General Linear Model (GLM) and Independent Component Analysis (ICA) (Friston et al., 1995; Beckmann and Smith, 2004). Graphs, or networks, are an intuitively appealing data structure for modeling the functional connectivity of the human brain. Nodes in a functional network represent predefined parts of brain tissue, and edges between nodes represent temporal correlations of their activity. The field of graph theory has introduced a range of metrics which, when calculated upon a network, are intended to provide insight into the topology or structure of that network. Many recent studies have applied such metrics to functional networks derived from brain imaging data, with the aim of discovering more about how the brain works, and how these metrics may vary across different groups of people. The results obtained by these studies (e.g., Zhao et al., 2012; Jacobs et al., 2013; Tijms et al., 2013) demonstrate the potential of complex network measures to act as biomarkers for a range of neurological disorders.

Relatively little research has been undertaken in exploring how these properties of functional networks change with aging and AD. Studies which explore changes to complex network measures such as mean path length and clustering coefficient are inconsistent in their findings, with some studies reporting AD-related increases, others reporting declines, and yet more reporting no change (Tijms et al., 2013). Furthermore, there are few prior studies which specifically look at the functional connectivity, or functional network properties, of aging with respect to a sensorimotor task and, again, there is little consistency in the findings of those studies which have explored this area.

In a review of studies concerning resting state functional connectivity in healthy aging, Ferreira and Busatto (2013) concluded that the most consistently identified age related changes to resting state connectivity are declines within and between regions of the default mode network, salience, attention, and motor networks, and connectivity increases in the prefrontal and frontal regions. Taniwaki et al. (2007) observed an age-related decrease in functional connectivity between the basal ganglia, thalamus, motor cortices and cerebellum in a hand movement task. Wu et al. (2007) observed age-related declines in connectivity in pre-motor and motor regions, in a simple finger tapping task. Agosta et al. (2010) found amnestic MCI and AD-related declines in connectivity between the sensorimotor cortices. Yan et al. (2011) identified age-related reductions in connectivity of the visual cortex during a visual task. Tomasi and Volkow (2012) observed an age-related increase to degree in the left and right motor cortices.

The default mode network (DMN) has traditionally been found to exhibit connectivity declines alongside the presence of AD (e.g., Greicius et al., 2004; Rombouts et al., 2005; Zhou et al., 2010; Petrella et al., 2011). Stam et al. (2006) and Wang et al. (2007) observed connectivity increases within the parietal regions, and Wang et al. (2007), Supekar et al. (2008), and Sanz-Arigita et al. (2010) detected a connectivity increase in the frontal and prefrontal regions. Wang et al. (2007) observed widespread AD-related reductions in functional connectivity associated with resting state fMRI. Stam et al. (2007) (see also Stam et al., 2009) found longer path length and reduced clustering coefficient in resting state EEG and MEG data acquired from individuals suffering from AD. Supekar et al. (2008) found AD-related increases in functional connectivity within the prefrontal and frontal regions, and reductions elsewhere. Similar trends were seen by Sanz-Arigita et al. (2010), who found AD-related declines in functional connectivity between the frontal, parietal and occipital regions, but increases within the frontal region. Wu et al. (2011b) identified widespread declines in hippocampal connectivity related to the presence of AD. Liu et al. (2014) observed AD-related reductions in functional connectivity within posterior regions and between posterior and anterior regions, and an increase in connectivity within the medial prefrontal cortex.

The variable nature of these findings leads us to claim that these properties of functional networks have not convincingly been shown to be able to reliably distinguish between the brains of healthy young and aged individuals, and of individuals suffering from Alzheimer's Disease. As the PASA phenomenon is a global pattern of change which has been reliably detected using traditional activation-based methods of fMRI analysis, it is arguably an ideal benchmark against which to test this claim. We hypothesize that the PASA phenomenon should be observable in the properties of functional networks, and argue that the confirmation of this hypothesis would provide evidence that functional network properties can be used to find consistent and reliable changes in brain architecture, related to aging and the presence of AD.

Functional network analysis, as applied to fMRI data, most often uses regionally averaged time series as the basis for network nodes, where each fMRI voxel is labeled according to an anatomical labeling scheme such as the AAL (Tzourio-Mazoyer et al., 2002) or LPBA40 (Shattuck et al., 2008) atlases (e.g., Supekar et al., 2008; Wang et al., 2010). An alternative approach is to represent every voxel as a node in the network, and to define network edges by calculating temporal correlations between the time series for every pair of voxels (e.g., Eguíluz et al., 2005; Markošová et al., 2009; Buckner et al., 2009). This voxelwise approach is more computationally expensive, but allows for more fine grained analysis of functional network properties (Hayasaka and Laurienti, 2010). This choice is also in agreement with the conclusion of a recent review on pros and cons of different definitions of nodes, in which the authors argue that the smallest possible subdivisions will yield the most unbiased and informative results (Stanley et al., 2013). Critically, no studies to date have attempted to identify AD-related changes in the properties of *voxelwise* functional networks derived fMRI data, and thus our work aims to tackle this gap in knowledge.

While some advances have been made in developing a framework for the analysis of global and regional functional network properties (e.g., Hosseini et al., 2012), there is no single standard approach to voxelwise functional network analysis, and a number of subtle issues arise when functional network analysis is applied to voxelwise neuroimaging data. In particular, while spatial normalization is a necessary step in group analyses of neuroimaging data, little research has been undertaken to explore its effects in a functional network analysis. We therefore provide an initial investigation into its effects.

The present study is based upon fMRI data originally collected by Buckner et al. (2000) (data set #2-2000-118W, fMRIDC, 2011). There were three main outcomes of their study. First was the finding of increased haemodynamic response amplitude in the visual region, and no difference in the motor region, of young adults when compared to aged adults, in association with performance of a simple visual motor task. Second was the observation of increased haemodynamic response variance in the aged with AD group relative to the other groups. Third was the finding that linear summation of the haemodynamic response, due to stimuli presented in rapid succession, is not affected by age or the presence of AD, as it was clearly seen in all three groups.

The primary aims of our present work are (1): to explore whether the PASA phenomenon can be observed in the properties of voxelwise functional networks derived from fMRI data; (2): to see how, if at all, any observed changes due to the PASA phenomenon are altered by the onset of clinically assessed AD; and (3): to assess the effect of spatial normalization order upon a functional network analysis.

## 2. Materials and methods

### 2.1. The data set

Structural and functional MRI data were acquired from 41 subjects: 14 young (9 females/5 males, mean age 21.1, SD 2.0), 15 healthy aged subjects (9 females/6 males, mean age 75.1, SD 6.9), and 12 aged subjects (7 females/5 males, mean age 77.1, SD 5.3) who had been clinically diagnosed with Dementia of the Alzheimer Type (DAT). These three groups shall be referred to as **young**, **aged**, and **aged with AD** respectively. The individuals in the aged and aged with AD groups were clinically assessed for the presence of dementia using the Clinical Dementia Rating, with all individuals in the aged group scoring CDR 0. Of the 12 individuals in the aged with AD group, seven scored CDR 0.5, corresponding to a diagnosis of probable AD; the remaining five scored CDR 1, corresponding to a diagnosis of mild AD.

Structural MRI images consisted of 128 × 1.25 mm slices, with each slice containing 256 × 256 1 mm^2^ in-plane isotropic voxels; these images were resampled to 128 × 128 × 75 2 mm^3^ isotropic voxels. Each functional MRI image consisted of 16 8 mm slices, acquired parallel to the anterior-posterior commissure, with each slice consisting of 64 × 64 3.75 mm^2^ in-plane isotropic voxels. Functional image slices were acquired in an interleaved manner, from superior to inferior, with even slices acquired first (Snyder, 2011, Private Communication). Functional image acquisition (TR) time was 2.68 s.

The study involved subjects completing a simple visual motor task within an event based experimental paradigm. Each subject underwent four fMRI recording sessions; within each session, 128 fMRI images were acquired over a period of 5 min, 43 s. During a single session, 15 trials were executed, with each trial consisting of either one or two visual stimuli, a checkerboard pattern flickering at 8 Hz, displayed for 1.5 s. The subjects were instructed to push a button with their right index finger upon onset of each stimulus. During a “one-stimulus” trial, the stimulus was triggered at the start of the trial. During a “two-stimulus” trial, the first stimulus was triggered at the start of the trial, and the second stimulus was triggered 5.36 s (2 image acquisitions) after the first. One- and two-stimulus trials were pseudorandomly intermixed. Each trial had a duration of 21.44 s (8 image acquisitions), the first trial in each session began 10.72 s after the beginning of image acquisition (at image #5), and the last trial ended at 5 min, 32 s (at image #124).

Some discrepancies regarding the classification of healthy aged subjects, and subjects diagnosed with AD, are apparent in the original study. One subject was listed as having scored CDR 0 in the assessment for DAT, but was subsequently placed in the aged with AD group. Another subject scored CDR 0, and was correctly placed in the aged group, but performed very poorly during the experiment in both reaction time and misses. For this analysis, we moved the former subject from the aged with AD group to the aged group, but left the latter subject in the aged group.

We chose the data from the Buckner et al. (2000) study as the basis for our own analysis for three reasons. First, the data set is publicly available due to the efforts of the fMRI Data Center (Horn and Gazzaniga, 2012). Since its initial publication, and the publication of the results of the original study, the data set has subsequently been used in numerous studies, and has generated a wide range of findings (e.g., Greicius et al., 2004; Tripoliti et al., 2010; Çiftçi, 2011). Second, this data set contains data from healthy young and aged individuals, and individuals diagnosed with probable to mild DAT, allowing us to achieve our aims of exploring changes due to age and the presence of AD. Finally, the study made use of a simple sensorimotor task, which allows us to explore age and AD-related changes in both sensorimotor activity, and in background activity which persisted throughout the duration of the experiment.

We accomplished this by splitting our analysis into two parts. In the first part, we analyzed fMRI data created by taking the average of every trial for each subject, in a similar manner to the analysis of Buckner et al. (2000). This analysis focuses upon the task related connectivity which was present within a single one- or two-stimulus trial, and is hence referred to as the **task based** analysis. The second part of the analysis, instead of focusing on task related connectivity, focuses upon functional connectivity which persisted throughout the duration of the experiment, thus reflecting background, or resting state connectivity. This part is hence referred to as the **resting state** analysis, and was made possible by concatenating the data acquired for each session into a single long fMRI volume. Justification for the validity of this approach is provided by Greicius et al. (2004) who used the same technique upon the very same data set to identify AD-related changes in default mode network activity.

The work of Dale and Buckner (1997) showed that the haemodynamic responses to multiple events occurring rapidly in succession summate in a linear manner. We therefore reasoned that, for the purposes of our task based analysis, there was no need to discriminate between one- and two-stimulus trial types. To further validate this approach, we performed a GLM activation analysis upon the task based fMRI volumes for every subject, and then combined the parameter estimates for each subject to estimate the extent of task related activity in each group. These results were compared with the results in the original study by Buckner et al. (2000), and are presented separately (McCarthy, 2014, Appendix A).

### 2.2. Processing

Before any preprocessing, the first four and last four images from every volume were discarded. Visual inspection of each volume uncovered three suspect data sets, all from the aged group. The data for two subjects contained aliasing effects, which were manually corrected. The data for one other subject were discarded, due to the presence of significant noise throughout every session. Thus, the number of individuals in the aged group was reduced to 14 (9 females/5 males, mean age 74.9, SD 6.9).

Every fMRI volume was corrected for slice timing differences, and a high-pass temporal filter applied with a pass frequency of (1/42.88 ≈ 0.02 Hz) (van den Heuvel and Hulshoff Pol, 2010). Motion correction was applied using FSL (Jenkinson et al., 2002). Non-brain matter (e.g., skull tissue) was removed from the structural MRI images, which were corrected for bias field inhomogeneities, and each MRI voxel classified as white matter, gray matter, or cerebrospinal fluid (Shattuck et al., 2001; Shattuck and Leahy, 2002). MRI and fMRI images were then registered to the 2 mm^3^) MNI152 T1 standard brain template (Andersson et al., 2007). No spatial smoothing was applied to the fMRI data, to avoid the introduction of artificial correlations between adjacent voxels (van den Heuvel et al., 2008; Hayasaka and Laurienti, 2010).

From the four session fMRI volumes for each subject, two fMRI volumes were created for analysis: a task based volume, and a resting state volume. The task based volume was created by calculating the average time series from all 60 trials across the four sessions. This resulted in a volume of 21.44 s duration (8 image acquisitions). The resting state volume was created by concatenating each of the four sessions into a single long volume of 21 min, 26.4 s duration (480 image acquisitions). A binary mask image, derived from the MRI tissue classification image, was used to mask all fMRI voxels that had not been classified as white matter or gray matter.

In order to explore the effects of spatial normalization upon functional network analysis, copies of both the task based and resting state volumes were transformed from the subject's native space into MNI152, or standard space. These standard space volumes were then resampled and interpolated back to the original native space resolution of 64× 64× 16 voxels. Functional network analysis was then performed on all four analysis volumes: the task based volumes in both fMRI and standard space, and the resting state volumes in both spaces.

A correlation matrix was created for each analysis volume, by calculating the Pearson correlation coefficient between all pairs of included voxels. Three unweighted and undirected networks were created from each of the task based and resting state correlation matrices, according to two-tailed (uncorrected) statistical significance thresholds of α = [1 × 10^−2^, 1 × 10^−3^, 1 × 10^−5^] for task based networks, and α = [1 × 10^−25^, 1 × 10^−38^, 1 × 10^−58^] for resting state networks. These significance thresholds respectively correspond to *r* ≈[0.82, 0.90, 0.96] and *r* ≈[0.45, 0.54, 0.63], and were empirically selected to ensure that the resulting networks were of a density suitable for analysis. Only positive correlations meeting or exceeding each threshold were included in the resulting networks.

Finally, disconnected nodes and small isolated components were removed from each network. This step was performed to prevent biasing of voxelwise network measures. For each network, any disconnected nodes were removed. Then, if the remaining network consisted of one major component which constituted at least 75% of the total network size, all other minor components were removed. For more fragmented networks consisting of multiple components, with no single major component, only the disconnected nodes were removed. These fragmented networks were excluded from statistical analysis of some network measures, in order to avoid bias due to disconnectivity.

Network measures calculated at every node in each network included degree, clustering coefficient and path length (Watts and Strogatz, 1998), and local efficiency (Latora and Marchiori, 2001). The degree of a node is the number of edges incident upon that node or, more simply put, the number of neighbors of that node. The clustering coefficient is the ratio of the number of edges which are present between a node's neighbors to the number of possible edges. In other words, the clustering coefficient of a node is the density of the subgraph formed by the node's immediate neighbors, and the edges which exist between them. The characteristic path length (or simply the mean path length) of a node is the average shortest path length from that node to all other nodes in a graph; the shortest path between two nodes is the minimum number of edges which must be traversed in order to join the nodes. Finally, the local efficiency of a node is the inverse of the mean path length of the subgraph formed by the neighbors of that node. Disconnected networks were excluded from the analysis of clustering coefficient and path length. Each node in a network represents a voxel located in the space of the underlying fMRI data (in either fMRI or standard space). Therefore, three dimensional images of these complex network measures were created, and formed the basis for the statistical analysis.

### 2.3. Statistical analysis

Properties of all functional networks were compared across each of the young, aged, and aged with AD groups, in order to find differences related to age and the presence of AD. Statistical analysis was performed against the null hypothesis of no difference, in any functional network properties, between each pair of groups. Where a disconnected network was excluded from analysis of a specific network measure, the degrees of freedom used in statistical calculation were adjusted accordingly. For each subject, voxelwise network measure images were normalized to *Z* scores in order to account for the effects of varying network size and density across subjects (Buckner et al., 2009; Sepulcre et al., 2010; Fransson et al., 2011). fMRI space images were transformed to MNI152 space, and masked using each subject's binary mask image (also transformed to MNI152 space using nearest neighbor interpolation) before voxelwise analysis proceeded. This transformation step was not necessary for standard space images.

Voxelwise network measure images were compared between each pair of groups using nonparametric cluster size thresholding (Hayasaka and Nichols, 2003; Winkler et al., 2014). Student's *t*-test was used to create an image of *t*-values upon the network measure images from each pair of groups. This *t* image was subjected to cluster size thresholding using a *t* threshold of 3.0. Then, *t* images were generated from 10,000 random permutations of group labels, and a maximal cluster size distribution created. The thresholded clusters in the observed *t* image were declared as significant if their size was in the top 95th percentile of the maximal cluster size distribution (i.e., a cluster level significance of α = 0.05). Use of the maximal cluster size distribution ensured control of the Familywise Error Rate (FWER). For a voxel to be included in a statistical test between two groups, a value had to be present at that voxel for at least 90% of all subjects from both groups. In other words, if more than 10% of subjects were missing a value at a particular voxel, that voxel was excluded from analysis.

In order to overcome the problem of missing voxels, either due to their representing disconnected nodes in the underlying functional network, due to subject specific atrophy, or to poor spatial overlap, values at missing voxels were imputed using a process adapted from the work of Vaden Jr et al. (2012), and referred to as *mean replacement by random neighbor selection*. This imputation process was applied before the statistical test described above. For a missing value in a subject's network measure image, a replacement value was generated by randomly selecting the values of up to 5 voxels from the images of all other subjects in the same group, located within a sphere of radius 10 mm, centered at the missing voxel. The mean of these sampled voxels was used as the replacement value. Imputation only proceeded if 90% of the randomly sampled voxels were present (i.e., the sampled voxels were not also missing values). After imputation, any missing voxels which could not be imputed were simply left as “missing”. This process was only applied to standard space network measure images, as it was deemed unnecessary for fMRI space images due to the spatial smoothing inherent in the image transformation process.

After statistical analysis had been performed on both fMRI and standard space functional networks, the level of correspondence between results from the two spaces was qualitatively assessed using two techniques. First, the Pearson correlation coefficient was calculated between fMRI and standard space statistical *t* images. Then we adapted Dice's Similarity Coefficient (Dice, 1945; Zou et al., 2004) to measure the extent of spatial overlap between significant voxels found in fMRI and standard space images. This adaptation is described separately (McCarthy, 2014, Appendix C).

## 3. Results

**Figure 1:**
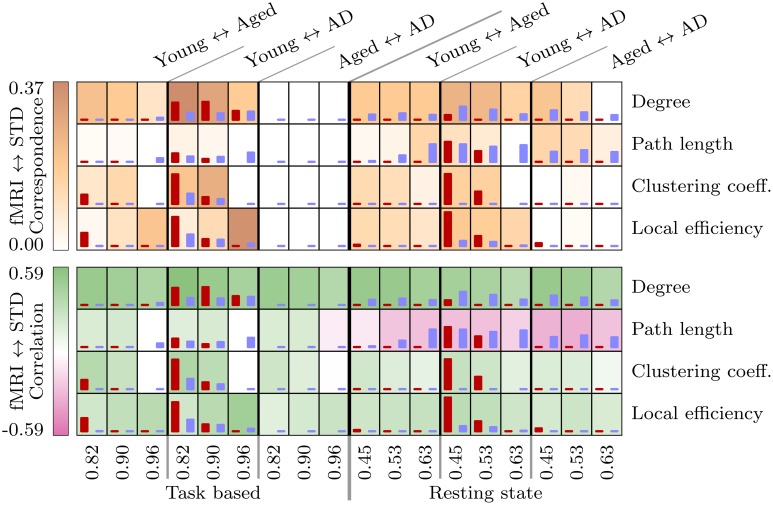
**These tables provides a summary of the statistical analysis upon voxelwise network measures between each pair of groups, in the task based and resting state networks**. The height of the red and blue bars are proportional to the number of significant voxels found in fMRI and standard space networks respectively, ranging from 0 to a maximum of 3085 voxels (resting state local efficiency, young ↔ aged with AD, threshold 0.45). The background shading at each test in the top table is proportional to the spatial correspondence of significant voxels, and in the bottom table to the Pearson correlation coefficient calculated between fMRI and standard space *t* images.

Results of the statistical analysis are summarized in Figure 1. Widespread differences, consistent across fMRI and standard space, were observed between the young group and the two aged groups in both task based and resting state networks. Differences related to the presence of AD were less widespread, with consistent differences between the aged and aged with AD groups only observed in resting state network measures. In order to identify the most consistent results across fMRI and standard space, and across correlation thresholds, we first identified voxels in which a significant group difference was found over at least two correlation thresholds. Then we used the LPBA40 anatomical atlas (Shattuck et al., 2008) to identify regions which contained significant group difference in the same direction, in both fMRI and standard space.

### 3.1. Age-related changes to task based networks

**Figure 2:**
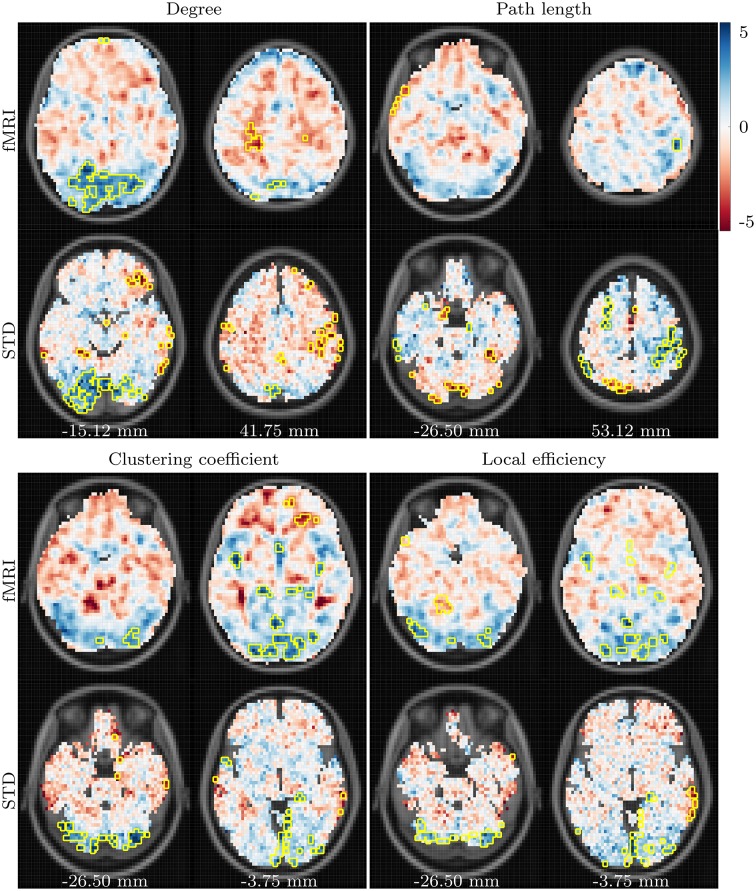
***t*-values showing differences between the young and aged groups in fMRI and standard space task based networks**. Data for degree, path length and clustering coefficient are shown for correlation threshold 0.82, and data for local efficiency shown for correlation threshold 0.96. Voxels found to be significantly different for at least two correlation thresholds are highlighted with a yellow border. Anterior is toward the top of the page, and subject left is to the left. Images and data are in MNI152 coordinate space. All data are overlaid upon the corresponding slice from the MNI152 template, resampled to the data resolution of 64 × 64 × 16 voxels. The values listed along the bottom specify the distance, in millimeters, of the displayed axial slices from the MNI152 origin, located at the anterior commissure.

Differences which arose between the young and aged groups are depicted in Figure 2. A clear pattern of reduced degree is present in the aged group when compared to the young group, centered around the lingual and inferior occipital gyri of both hemispheres. The aged group also exhibits a tendency toward increased bilateral degree in the pre- and post-central gyri. Both of these differences are present across all correlation thresholds.

Results for path length demonstrate the lowest level of consistency across fMRI and standard space functional networks. The most consistent age-related difference observed for path length is a bilateral reduction in the pre- and post-central gyri.

Age-related differences in clustering coefficient closely resemble those seen in degree, with age-related declines in bilateral posterior regions, predominantly in the lingual gyri, cerebellum and cuneus. However, unlike the results for degree, the strength of these differences weakens as the correlation thresholded is increased, and the consistency of significant results across threshold and across fMRI and standard space is relatively low. Finally, age-related declines in local efficiency are present bilaterally in the lingual and occipital regions. In contrast to the results for degree and clustering coefficient, consistency between fMRI and standard space results for local efficiency increase as the correlation threshold is raised.

These trends are clearer when the results are viewed in a regional manner, as shown in **Figure 5**. There is a clear pattern of age-related reductions in degree, clustering coefficient and local efficiency in posterior regions of the brain when the young and aged groups are compared. This is accompanied by age-related increases across the same network measures in more anterior regions.

### 3.2. Age-related changes to resting state networks

**Figure 3:**
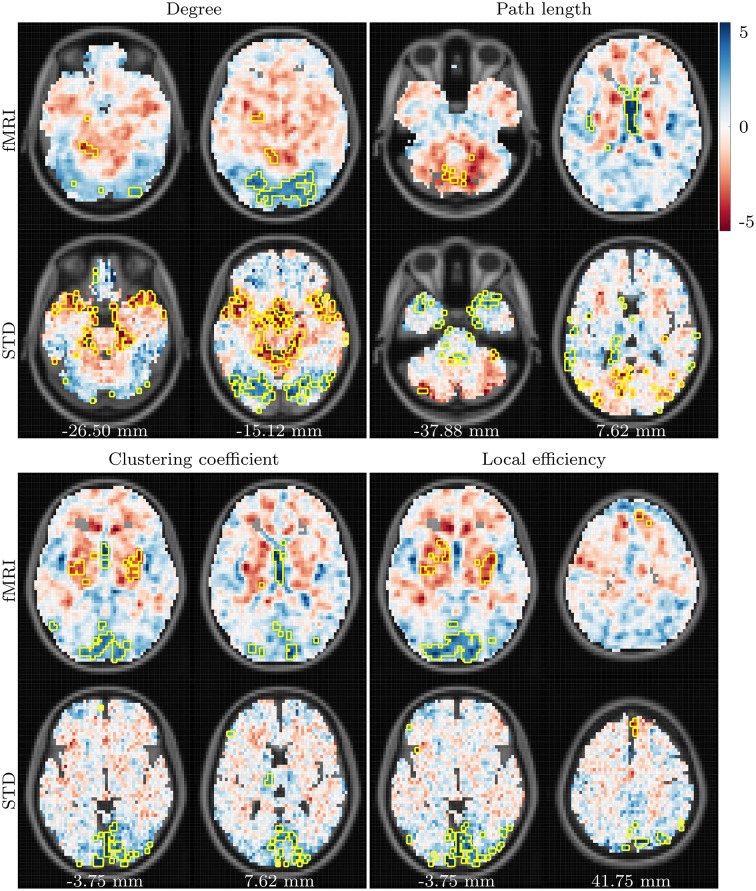
***t*-values showing differences between the young and aged groups in fMRI and standard space resting state networks**. Data for all network measures are shown for correlation threshold 0.45.

Age-related differences observed in resting state networks are shown in Figures 3, **6**. In a similar manner to the task based networks, an age-related decline in degree is present bilaterally in posterior regions, primarily the occipital lobe, lingual gyrus and cuneus. An age-related increase in degree is also present in the cerebellum, and in the left hippocampal and parahippocampal regions.

Results for resting state path length again demonstrate poor consistency across fMRI and standard space, with *t* images even demonstrating a tendency toward negative correlatedness (recall Figure 1). Age-related differences in path length, which are consistent across spaces and correlation thresholds, are centered around an age-related reduction in the left superior temporal gyrus and the caudate, and age-related increases in the cerebellum.

In a similar manner to the task based networks, age-related changes to resting state clustering coefficient are similar to those for resting state degree. The strongest difference in clustering coefficient between the young and aged groups is an age-related reduction in the occipital region, cuneus and lingual gyri of both hemispheres. Similar patterns are present for local efficiency, with the aged group exhibiting reduced local efficiency in the same regions, alongside increased local efficiency in the superior frontal gyrus. Once again, these results are highlighted when viewed regionally, as depicted in **Figure 6**.

### 3.3. AD-related changes to resting state networks

**Figure 4:**
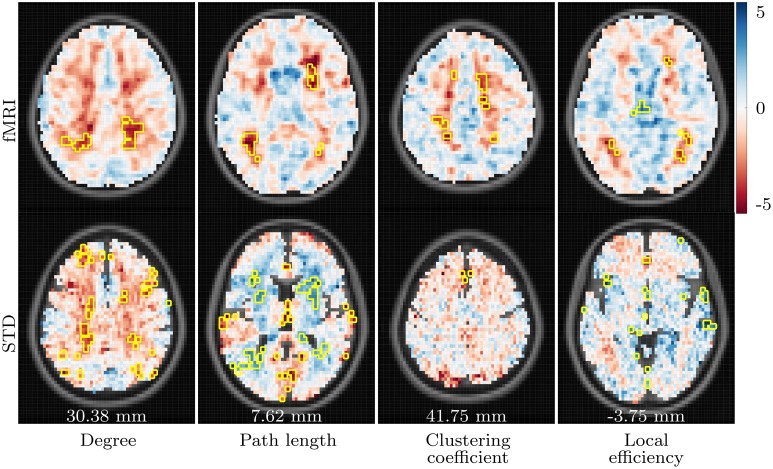
***t*-values showing differences between the aged and aged with AD groups in fMRI and standard space resting state networks**. Data for all network measures are shown for correlation threshold 0.45.

AD-related differences observed in resting state networks are shown in Figures 4, **6**. Consistency between the results for fMRI and standard space networks is quite poor overall, however some differences do stand out. The aged with AD group demonstrates increased degree, in comparison to the aged group, in the left angular gyrus, and bilaterally in the superior parietal gyrus. Results for path length are again highly inconsistent, with the only result that occurred in both fMRI and standard space networks an AD-related increase in path length in the left middle temporal gyrus.

Finally, the aged with AD group demonstrates increased clustering coefficient in the superior frontal gyrus of both hemispheres, and reduced local efficiency in the brainstem, when compared to the aged group.

## 4. Discussion

**Figure 5:**
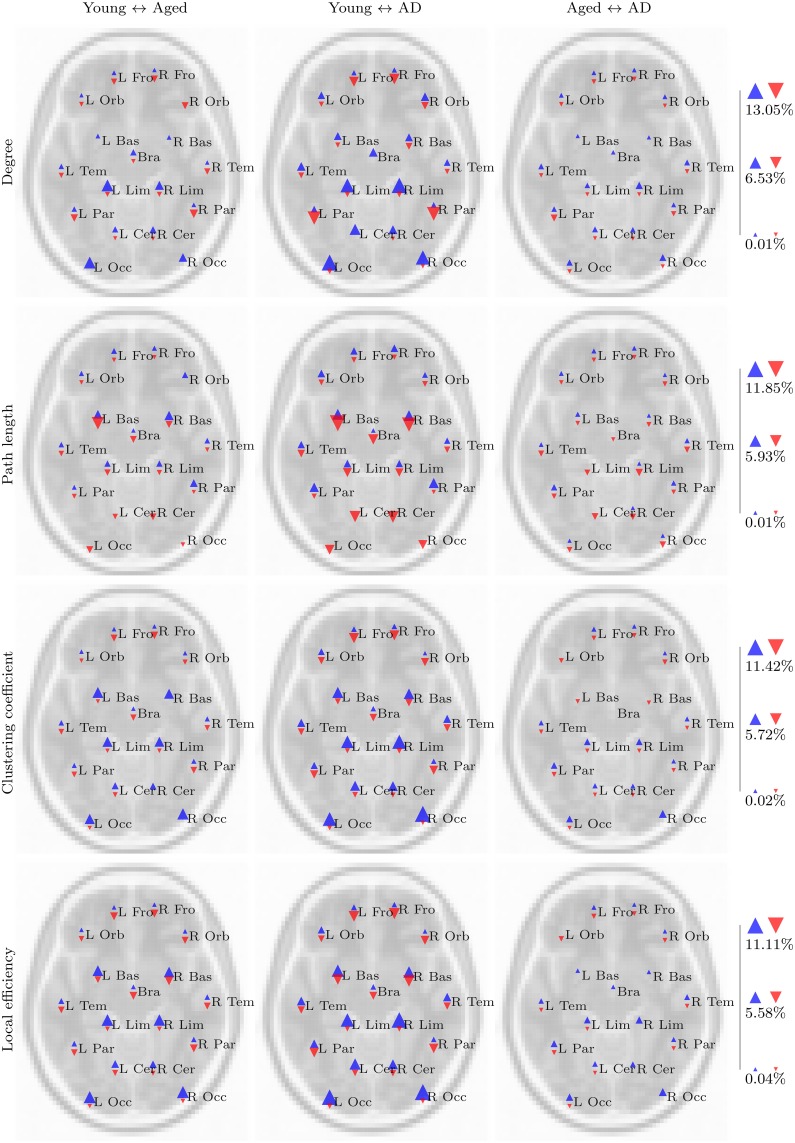
**Summary of regional group differences in task based networks**. The images display the total proportion of voxels for each region, normalized by region size, in which a significant difference in the respective network measure was observed between each pair of groups, in both fMRI and standard space, across all correlation thresholds. The region labels are based upon a reduced version of the LPBA40 anatomical atlas (Mazziotta et al., 2001; McCarthy et al., 2013). Blue triangles pointing upwards reflect the proportion of voxels in which a significant increase in the respective network measure (e.g., degree) was observed in the first group, relative to the second group (e.g., the young group, for the young versus aged test). Similarly, red triangles pointing downwards reflect the proportion of voxels in which a significant decrease was observed in the first group relative to the second group. The triangles are scaled separately within each row, so their sizes are comparable across group pairs for each network measure.

**Figure 6:**
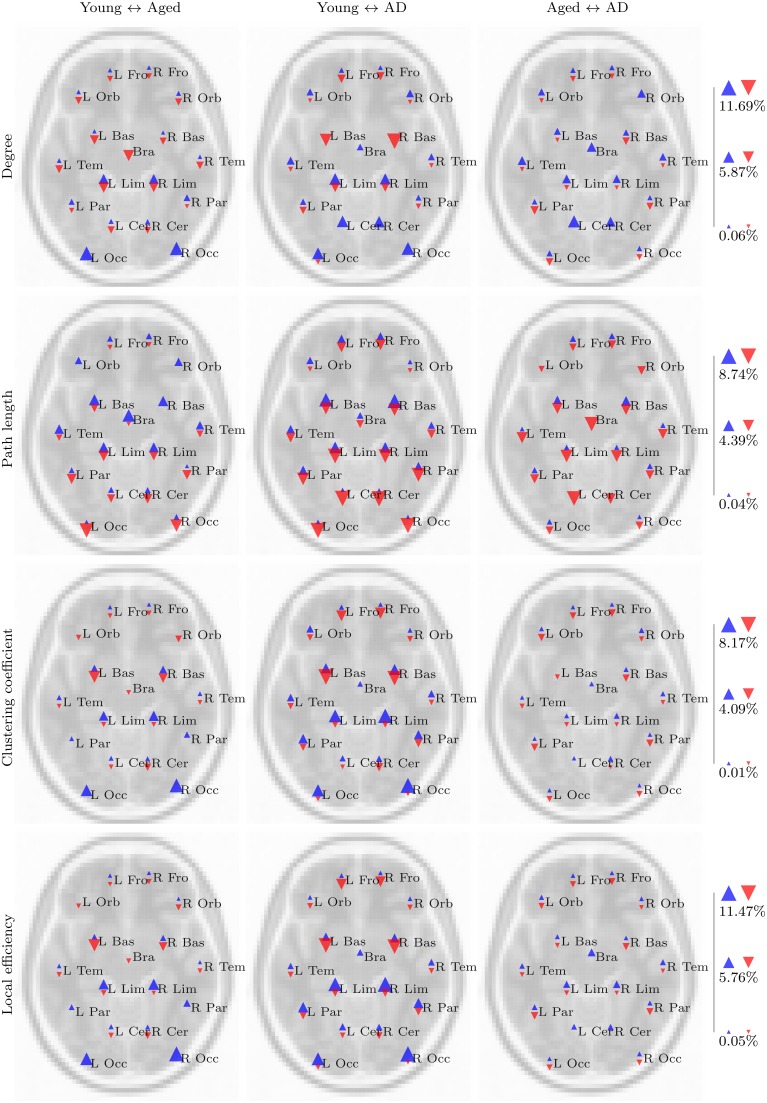
**Summary of regional group differences in resting state networks**.

Our aims in this analysis were to explore whether the PASA phenomenon could be observed in the properties of voxelwise functional networks; to see whether these observations were changed in any way by the presence of AD; and to assess the effect of spatial normalization order upon the results of a functional network analysis. Clear and consistent differences were present when the young and aged groups were compared, with the strongest differences being age-related declines in degree, clustering coefficient and local efficiency in posterior regions coupled with increases in more anterior regions, in both task based and resting state networks. On the whole, these differences were amplified by the presence of AD, as is evident in the regional analysis (Figures 5, 6). However, despite a number of trends in the results, there were few findings which consistently differentiated between healthy aging and cases of mild AD.

### 4.1. Age and AD-related changes to functional network properties

#### 4.1.1. Manifestation of PASA in functional network properties

The process of aging appears to have a substantial effect upon voxelwise functional network properties, with widespread differences observed between the young and aged groups in both task based and resting state networks. However, the effect of mild or very mild Alzheimer's disease on these same networks is more subtle, with weaker differences observed primarily in the resting state networks. A clear pattern of declining task related functional connectivity (as measured by node degree) in the aged group, when compared to the young group, is evident in posterior regions, most notably the occipital and lingual gyri. The reverse trend is present in more anterior regions, centered around the pre- and post-central gyri of both hemispheres. These differences appear to be enhanced in the aged with AD group. Age-related changes to the other network measures in task based networks closely resembled those for degree, leading us to suspect that they were primarily driven by differences in node degree.

Age-related differences in the resting state networks resembled the changes that were observed in the task based networks, with age-related reductions to degree in posterior regions centered around the lingual gyri, cuneus, and occipital lobe. Alongside these declines in degree were declines in both clustering coefficient and local efficiency, in the same regions. Degree increases in the aged group, when compared to the young group, were present in the cerebellum, and the left parahippocampal and hippocampal regions, in addition to an increase in local efficiency in the superior frontal gyri. The observed age-related declines in posterior connectivity, and associated changes to the other complex network measures, are in agreement with the majority of studies exploring the effects of aging upon resting state functional connectivity and network properties (Ferreira and Busatto, 2013). The hippocampal and cerebellar degree increases observed in our results are more surprising, as these regions are generally associated with age-related connectivity declines. One study consistent with our findings is that of Tomasi and Volkow (2012), who also observed age-related degree increases in the hippocampal and cerebellar regions. These findings are also in agreement with more general observations of age-related overactivation in anterior regions, and underactivation throughout the rest of the brain (Rajah and D'Esposito, 2005; Rugg and Morcom, 2005).

The above findings imply that the properties of functional networks are strongly influenced by the PASA phenomenon. In both task based and resting state networks there are more voxels with reduced local efficiency, degree and clustering coefficient in posterior parts of the brain, primarily in the occipital gyri, of the aged group when compared to the young group. The aged group also exhibits an increase in the same network measures in anterior regions. These very same patterns are enhanced when the young and aged with AD groups are compared, lending evidence to the idea that the PASA phenomenon may also be present in early-stage AD. This is supported by numerous activation studies which have reported frontal over-activation in individuals diagnosed with AD (Flashman et al., 2003; Pihlajamäki and Sperling, 2011).

#### 4.1.2. AD-related changes to functional network properties

To our knowledge, this is the first study to explore AD-related changes to the properties of voxelwise functional networks derived from fMRI data. While the differences, in task based networks, observed between the young and aged groups appeared to be enhanced in the aged with AD group, this did not manifest as consistent significant differences between the aged and aged with AD groups. Some trends were however apparent in resting state networks, with AD-related degree increases in the left angular gyrus and superior parietal gyri, clustering coefficient increases in the superior frontal gyri, and local efficiency decline in the brainstem. With the exception of the angular gyri, these trends are consistent with the findings of other studies. The angular gyri are considered to be involved in the default mode network (DMN), which has traditionally been found to exhibit connectivity declines alongside the presence of AD (e.g., Greicius et al., 2004; Rombouts et al., 2005; Zhou et al., 2010; Petrella et al., 2011). However, these declines are usually centered around the posterior cingulate cortex (PCC) and the hippocampal formations, with the angular gyri receiving little attention. Furthermore, a recent study by Damoiseaux et al. (2012) suggests that AD-related changes in DMN activity are not uniform, with connectivity increases and declines throughout different regions of the DMN. No studies to date have reported AD-related changes to functional network properties in the brainstem.

The regional summaries shown in Figure 6 indicate AD-related decreases in degree and increases in path length, throughout posterior brain regions. This is indicative of a disruption in functional connectivity in the brains of individuals suffering from AD, and is consistent with the majority of previous studies which have explored the functional connectivity and functional network properties of AD. Furthermore, despite few significant differences being uncovered in the statistical analysis, Figures 5, 6 do reveal some trends which suggest that the PASA phenomenon may be accentuated by the presence of AD. In particular, the differences which are present between the young and aged groups appear to be stronger when the young and aged with AD groups are compared. These trends also appear to be present when the aged and aged with AD groups are compared; however a statistical analysis with larger sample sizes, and hence more statistical power, would be required to determine if these trends reflect real differences related to the presence of AD.

### 4.2. Methodological issues

#### 4.2.1. Complex network properties

**Figure 7:**
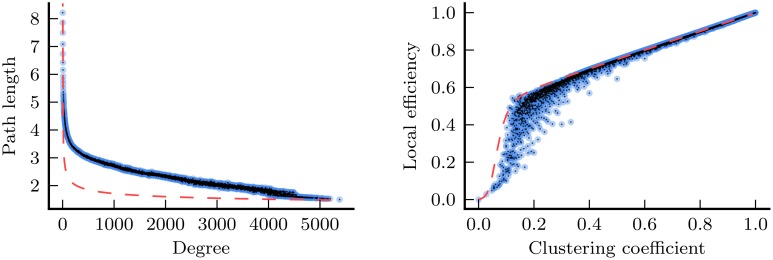
**Relationships between degree and path length (left), and between clustering coefficient and local efficiency (right) for the nodes from every functional network in the study (joint histograms with 5000 bins, based upon data from a total of 275 networks/3489756 nodes; 5 networks in the study contained more than one component, thus were excluded from these plots)**. Data are shown before *Z*-normalization was applied. For the purposes of comparison, the dashed red lines show the same relationships for Erdős-Rényi (ER) random graphs (Erdős and Rényi, 1960). In an ER graph of size *n*, the mean path length, *l*, may be derived from the mean network degree, *k*, according to $l=\frac{\ln[n]-0.5772}{\ln[k]}+\frac{1}{2}$ (Fronczak et al., 2004). For the left plot a value of *n* = 7769, the mean number of nodes across all networks, was used. Furthermore, in an ER graph, mean local efficiency, *e*, may be derived from mean clustering coefficient, *c*, according to the equation $e=wc+\frac{x}{1+{\left(c/y\right)}^{z}}$ for constants *w* ≈ 0.5, *x* ≈ 0.5, *y* ≈ 0.07, and *z* ≈ −3.9 (McCarthy, 2014).

In this study, we have explored the manifestation of the PASA phenomenon in the functional network properties of healthy young and aged individuals, and individuals diagnosed with AD. This avenue of research is ultimately based upon the hypothesis that the topology of functional networks reflects, albeit in a complex and nonlinear way, the underlying physical and functional relationships in the brain. Given that the PASA has been consistently observed in activation-based studies, our own detection of the PASA phenomenon in the functional network properties covered in our analysis lends support to this hypothesis. Qualitative inspection of our results identified some general relationships that exist between these network properties. For instance, we identified strong consistencies in the results between degree and path length, and also between clustering coefficient and local efficiency. Figure 7 shows that there are strong relationships present between both pairs of measures in the underlying network data. These relationships lead us to conclude that, for the purposes of group comparison, path length is likely no more than a proxy for degree, offering little new information. The same may be said for local efficiency, with respect to clustering coefficient.

#### 4.2.2. Spatial normalization order

There is no correct answer to the question of when spatial normalization should be applied during analysis of fMRI data. While the effects of spatial smoothing have been extensively studied (e.g., Hopfinger et al., 2000; Triantafyllou et al., 2006; Mikl et al., 2008; Carp, 2012), there are precious few studies that explore the effects of spatial normalization upon the results of fMRI analyses. Carp (2012) tested a large range of analytical fMRI processing pipelines in an activation analysis of a motor response inhibition task, including the application of spatial normalization both before and after GLM model estimation, but did not find a large effect of normalization order in the results. However, the findings of Wu et al. (2011a) suggest otherwise - they evaluated the order in which spatial normalization is applied to a seed based correlation analysis of resting state fMRI data, where the seed regions were identified using a prior activation analysis of a motor task. In that study, the authors did observe a large effect, of the spatial normalization order, upon the analysis results.

**Figure 8:**
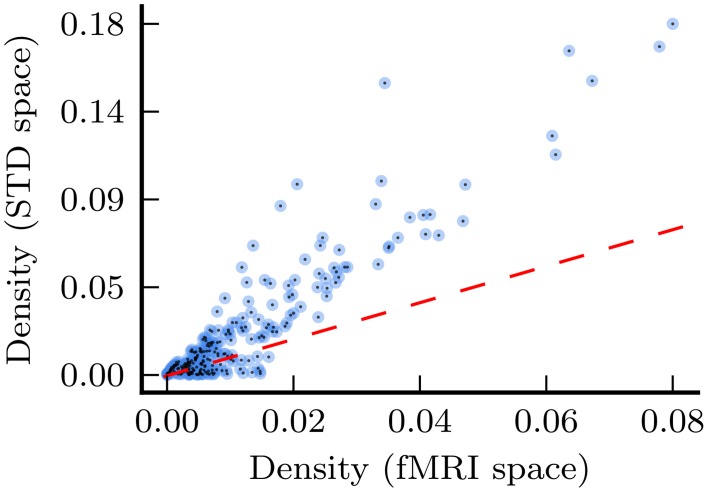
**Density for every network in the analysis, with density for fMRI space networks plotted against density for the corresponding standard space networks**. The dashed red line shows the relationship *x* = *y* for the purposes of comparison.

The results presented in this study demonstrate that the point at which spatial normalization is applied does have a substantial effect upon functional network analyses. The most noticeable effect is to network density, as depicted in Figure 8: in general, standard space networks have substantially higher density than the corresponding fMRI space networks. We speculate that this effect is due to artificial spatial and temporal correlations introduced to the fMRI time series, by the spatial warping and smoothing which is inherent in the spatial normalization process.

Another issue related to spatial normalization was the presence of disconnected nodes in fMRI space networks, which had an effect upon other network measures. Specifically, this issue was responsible for the negative correlation between *t*-values calculated upon fMRI and standard space resting state path length, as depicted in Figure 1. Once again, the smoothing process caused by spatial normalization complicates this situation, by smoothing the values of connected voxels into disconnected regions, with the effect that regions which were disconnected (i.e., missing) in fMRI space images, when transformed to standard space, were replaced with values at or near to 0. The disconnectivity problem did not affect standard space analysis because voxels representing disconnected nodes were either imputed or excluded from analysis. Furthermore, as described above (see Figure 8), standard space networks had higher density than their fMRI space counterparts. In the resting state networks, this translated to much lower levels of disconnectivity in standard space networks.

## 5. Conclusion

In this study, we performed a large scale voxelwise analysis of task based and resting state functional networks, with the aim of determining whether the PASA phenomenon, commonly observed in activation studies of aging, would be observable in the properties of functional networks. Our results strongly suggest this to be the case and, furthermore, suggest that the presence of mild AD amplifies the effect that PASA has upon functional network properties. These findings suggest that properties of functional networks have the potential to be used as biomarkers for the identification of neurological disorders such as AD. Finally, our results demonstrate that the point at which spatial normalization is applied in a functional network analysis has a substantial effect upon the results of such an analysis.

## Author contributions

Paul McCarthy, Lubica Benuskova, and Elizabeth A. Franz conceived and designed the experiment, Paul McCarthy performed the analysis and drafted the initial manuscript, and Paul McCarthy, Lubica Benuskova, and Elizabeth A. Franz revised and edited the manuscript.

### Conflict of interest statement

The authors declare that the research was conducted in the absence of any commercial or financial relationships that could be construed as a potential conflict of interest.

## Supplemental data

The presented work was performed as part of a larger study, the details of which, in addition to all associated functional network data, are provided in McCarthy et al. (2013).
